# Is There an Association between Sleep Disorders and Diabetic Foot? A Scoping Review

**DOI:** 10.3390/jcm10112530

**Published:** 2021-06-07

**Authors:** Raúl Fernández-Torres, María Ruiz-Muñoz, Ana J. Pérez-Belloso, Jerónimo García-Romero, Manuel Gónzalez-Sánchez

**Affiliations:** 1 Department of Nursing and Podiatry, University of Málaga, Arquitecto Francisco Peñalosa, s/n, Ampliación Campus de Teatinos, 29071 Málaga, Spain; raulft.95@gmail.com; 2 Department of Podiatry, Faculty of Nursing, Physiotherapy and Podiatry, University of Sevilla, 41009 Sevilla, Spain; aperez30@us.es; 3 Medical School of Physical Education and Sports, University of Málaga, C/Jiménez Fraud 10, Edificio López de Peñalver, 29010 Málaga, Spain; jeronimo@uma.es; 4 Department of Physiotherapy, University of Málaga, Arquitecto Francisco Peñalosa, s/n, Ampliación Campus de Teatinos, 29071 Málaga, Spain; mgsa23@uma.es

**Keywords:** diabetic foot, diabetic foot ulcer, diabetic neuropathy, sleep, obstructive sleep apnea

## Abstract

Diabetic foot is associated with a low quality of life since physical disabilities, mood disturbances and psychological disorders are frequent. One of the most important biological processes to ensure quality of life is sleep. Sleep disorders can impair glycemic control in patients with diabetes mellitus or even cause long-term type 2 diabetes mellitus. The aim of this study is to carry out a scoping review about the association between sleep cycle disorders and diabetic foot. PubMed, Scopus, CINAHL, PEDro, Cochrane Library, SCIELO and EMBASE databases were chosen for the search and the following terms were used: “diabetic foot”,“sleep*”,“rest-activity”,“mood” and“behavior”. All the studies should include outcome variables about sleep and diabetic foot. Finally, 12 articles were selected, all of whichwere observational. The most frequent variables were those regarding diabetic foot ulcer aspects and diabetic neuropathy on one side, and obstructive sleep apnea, sleep duration and sleep quality on the other side. The results suggest that there is a possible association between obstructive sleep apnea and the presence or history of diabetic foot ulcers. No direct associations between sleep quality or sleep duration and diabetic foot or diabetic foot ulcer variables have been found.

## 1. Introduction

In many cases, diabetes mellitus (DM) leads to several complications, of which diabetic foot (DF) is one of the most frequent [1]. It likely begins with the onset of diabetic neuropathy (DN) and peripheral arterial disease (PAD) [2].Unless this situation is prevented, it can result in diabetic foot ulcers (DFUs), which tend to become infected and show poor healing [3,4]. The risk of developing wounds is 25% higher in a patient with DM [5]. The most advanced stage of DFU often requires lower limb amputation, and this is an important source of diabetes mortality [6]. Along with the mortality and morbidity of DFUs, the economic consequences are high. In Europe, the cost of treating DFUs varies from approximately 4500 to 16,800EUR per patient [7].

DF is associated with a low quality of life, since physical disabilities and mood disturbances (among other situations) are frequent [8]. Psychological disorders, such as anxiety and depression, are also not uncommon [9]. Pain is another important factor affecting quality of life, caused by the existence of DFUs, DN and PAD symptoms [10,11], phantom limb syndrome [12] or the combination of all of these.

One of the most important biological processes to ensure quality of life is sleep, which can be altered due to sleep disorders, lifestyle, psychosocial and environmental factors, or medical conditions [13].

In fact, sleep disturbances are identified as a disruptive event that favors the appearance and chronification of pathologies. Furthermore, it has been identified that this disturbance is bidirectional, in the sense that the treatments carried out in patients with chronic pathologies have a lesser effect when they suffer from sleep disturbances [14]. Among the main effects that cause sleep disturbance are depression, fatigue, exhaustion, decreased quality of life and cardiac, systemic and metabolic alterations [14,15]. Specifically, in patients with DM, it has been observed that sleep disturbances cause alterations in glycemic control [16], a fundamental variable in the management of these patients, causing, in the long term, an increase in patients with type 2 DM [17].

Despite the fact that it has been shown that sleep disorders are directly and negatively related to the appearance, capacity for adaptation and response and possibilities of recovery from chronic diseases, to the best of our knowledge, there are no reviews published that study the association between sleep disorders and DF, either directly (due to patho-physiological reasons) or indirectly (due to psychogenic issues derived from the pathology).

The main objective of the present study is to carry out a scoping review of the literature about the association between sleep disorders and DF.

## 2. Materials and Methods

This review was carried out according to the guidelines and recommendations of the Preferred Reported Items for Systematic Reviews and Meta-Analysis (PRISMA) [18].

### 2.1. Search and Sources

PubMed (Medline), Scopus, CINAHL, PEDro, Cochrane Library, SCIELO and EMBASE databases were used. The following terms were used alongside “OR” or “AND”: “diabetic foot”,“sleep*”,“rest-activity”,“mood”,“behavior”.

The following database search strategy was used: ((((Sleep* [Title/Abstract]) OR Rest-Activity [Title/Abstract]) OR Mood [Title/Abstract]) OR Behavior [Title/Abstract]) AND (diabetic foot [Title/Abstract]).

### 2.2. Eligibility Criteria

The inclusion criteria were that studies should be observational, experimental or mixed. In these studies, the sample should consist of patients with DF. Age, sex and the type of diabetes of the sample were not considered. All documents published up to 30 July 2020 were included. The exclusion criteria were the absence of outcome variables about sleep or DF, and studies that were not conducted on humans. Documents not published in English, Spanish, German, French or Italian were also excluded.

### 2.3. Selection of Studies

Two independent researchers were involved in each stage of the study selection. Initially, a screening was carried out based on the title and abstract of the articles resulting from the search strategy, checking the contents of the full article if necessary. The articles were then evaluated for selection based on the previously mentioned eligibility criteria. Disagreement between the articles chosen by each reviewer was solved by the intervention of a third reviewer, who ultimately decided if a study was included or excluded.

### 2.4. Data Extraction and Synthesis of Results

In order to have a general approach to the studies, a Table 1 was designed to show their structural characteristics: authors, date of publication, type of study, sample size, type of diabetes, gender and age. In Table 2, the outcome variables regarding DF and sleep were presented. This table included information such as the characteristics of DFUs, DN, sleep quality, insomnia and breathing disorders, among others. These outcome variables were extracted to analyze how often were they were studied and to determine which were the most relevant, thus facilitating the comparison of results between studies.

After the extraction and comparison of the variables related to DF and sleep, Table 3 was constructed to show the associations found in the selected studies.

## 3. Results

### 3.1. Selection of Studies

**Figure 1:**
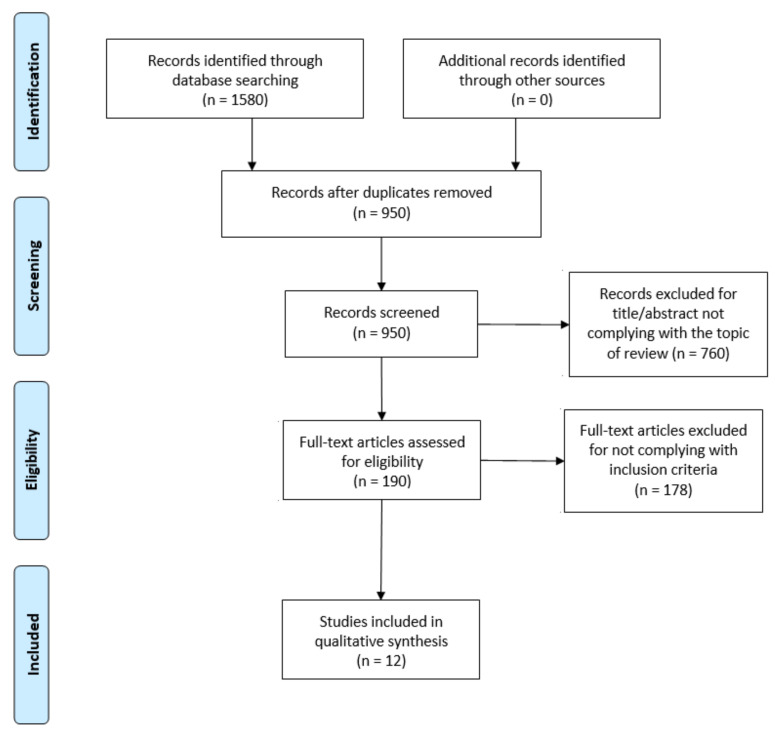
PRISMA flow diagram adapted with permission from The PRISMA group, 2020.

The flowdiagram (Figure 1) summarizes the study selection process, specifying the reasons for study exclusion. The main reason for exclusion was the absence of variables related to the sleep cycle or DF in the main objective of the study. There were 12 articles selected that were published since 2009 and included DF and sleep outcome variables.

All selected studies were observational, most of them cross-sectional (n = 6). The remaining studies were cohort studies (n = 2), case-control studies (n = 2), one case-report and one case series study. The sample size varied from n = 3 [19] to n = 1,656,739 [20], with the total sample size being n = 1,659,699. In 3studies [21,22,23], the type of diabetes in the sample was not specified. In 6studies [19,20,24,25,26,27], the sample subjects had type 2 diabetes mellitus (DM2), and in 3studies [28,29,30], they had both type 1diabetes mellitus (DM1)and DM2. In all of them, the population was adults(>18 years old) (Table 1).

### 3.2. Data Extraction and Synthesis of Results

The outcome variables analyzed in the studies were divided into DF-related and sleep-related. The most frequent variables in the first group were those regarding DFU aspects, followed by those related to DN. In the second group, the most frequent variables were those regarding obstructive sleep apnea (OSA), sleep duration and sleep quality (Table 2). All studies defined the instruments for measuring the outcome variables, except for the studies by Bener et al. [25] and Andruskiene et al. [21].

Table 3 shows a summary of the findings of the selected studies. All of them were observational, so there was no intervention to be considered and no quantitative analysis could be performed on the results. OSA showed an association with the development of DN and PAD, the severity of DFUs, the history of DFUs and healing capacity. Sleep quality was associated with the use of certain therapies for DFU healing and showed contrary associations with the presence of DFUs. Sleep duration was not associated with the presence of DFUs but was associated with the level of amputation.

## 4. Discussion

Obstructive sleep apnea (OSA) is a common sleep disorder in which a partial or complete obstruction of the upper airway occurs [31]. These obstructions will lead to a greater division of sleep, a decrease in oxygen saturation and a reduction in air flow [32]. OSA is an independent risk factor for cardiovascular disease [33], cognitive disorders [34] and metabolic dysfunction [35].Intermittent hypoxia increases sympathetic activation and oxidative stress, impairing arterial function and generating inflammation [36].

It is known that untreated OSA leads to morbidity and worsening of glycemic control (insulin resistance and glucose intolerance), along with diabetic angiopathy. Research has shown that OSA is related to insensitivity of the foot and diabetic peripheral neuropathy, all of which contributes negatively to DFU healing [37,38]. In four of the studies included, DF variables were associated with OSA-related variables. Altaf et al. [24] found a positive correlation between small fiber neuropathy and OSA severity, and between the prevalence of DFUs and OSA. This suggested that OSA patients should be considered to be high risk, although the sample size did not allow regression analyses. Subramanian et al. [20] recommended anticipating the development of OSA as a risk factor in patients with DF.

Maltese et al. [28] highlighted the high prevalence of OSA among patients with DFUs, concluding that OSA severity is directly related to poor healing and re-ulceration.They used the STOP-Bang Questionnaire for OSA severity, a widely used instrument with high levels of sensitivity and specificity [39]. Therefore, the presence and severity of OSA should be considered in the treatment and prevention of DFUs. As a matter of fact, Vas et al. [19] described three cases of patients with DM2 and obesity, in which they studied how the impact of severe OSA interfered with DFU healing, despite a good local treatment. Patients under OSA treatment with continuous positive airway pressure showed significantly improved DFU healing, while patients who refused OSA treatment did not improve. Despite sample limitations, the results were promising and could represent a breakthrough in DFU treatment in patients with similar characteristics.

In another four of the studies included, sleep quality was related to DFU-related variables. Haveleia and Gayatri [23] found a significant correlation between the levels of stress and pain and subjective sleep quality in DFU patients, although they found no relationship between the severity of DFUs and sleep quality. However, it must be taken into account that this study did not include a control group in the sample. Conversely, Salomé et al. [30] did provide results that supported DFU patients having poor sleep quality, although this study did not include a control group without DFUs. Puspita et al. [26] addressed this same relationship and included a control group of people with diabetes without DFUs. As in the previous study, most of the subjects with DFUs and/or pain had poor sleep quality, although no significant differences were found.

All of these studies used the Pittsburgh sleep quality index (PSQI) to measure sleep quality, which is valid, reliable and widely used [40]. This lack of relationship between sleep quality and the presence or severity of DFUs could be explained by the high probability of DFUs not causing any pain (because of DN) [41]. Moreover, not all DFU patients suffer from sleep disorders [42].

Nairetet al. [22] analyzed the effectiveness of microcurrent therapy for the healing of chronic ulcers (including DFUs), and one of the outcome variables was sleep quality. Both sleep quality and neuropathic pain improved in most patients after receiving the therapy, however, the sleep quality measurement instrument was not specified, nor was a control group used. These results should be taken with caution and the use of microcurrent therapy should be studied in detail in future works.

Another important variable regarding sleep is its duration, which was studied by Sheahan et al. [27] in patients with DFUs under different conditions, as follows: minor amputation, major amputation, with and without off-loading elements, or peripheral neuropathy. These groups were compared to each other and did not show a significant decrease in quality or duration of sleep. This lack of correlation can be explained in a similar way as before, in that the absence of pain and psychogenic or sleep disorders may be the reason for the sleep not being impaired.

The study by Andruskiene et al. [21] found an association between diabetic foot pain and depressive states in a female population, however, no correlation was found with variables regarding the sleep. According to other authors, the depressive state can cause sleep disturbances [43,44]. This study was the only one included in this review that took into account the consumption of sedative, antidepressant, analgesic or antitussive drugs.

In two of the studies included, DF and sleep variables were not related to each other. In the study by Bener et al. [25], sleep variables and DF variables were studied separately as risk factors for hearing loss. Rutkove et al. [29] also did not relate them. Instead, thermoregulation in the foot and DN during sleep and wakefulness were measured, and it was concluded that nocturnal thermoregulation is affected in patients with ND.

Along with our results, recent systematic reviews and meta-analyses show the association between DM on the sleep. Reutrakul et al. [45] found an association between DM1, poor sleep quality and prevalence of OSA, while another review [46] also associated the latter with DM2. Lee et al. [47] and Grandner et al. [48] found that the quality and duration of sleep influenced both glycemic metabolism in patients with DM2 and the risk of suffering from DM2. Several authors concluded that there is a high prevalence of sleep disturbances in patients with DM [35,49,50]. In the recent work by Nefs et al. [51], the reciprocal relationship between DM and sleep was approached from a behavioral science perspective, and it was stated that sleep quality should be considered with the same importance as diet and exercise in DM care.

Our results lead to several applications in clinical practice. Since the relationship between OSA and DF is the most studied, clinicians should consider OSA as a component of the multifactorial condition of DF. In addition, it may be appropriate for a diabetic foot specialist and a sleep disorder specialist to work in a multidisciplinary way with OSA and DF patients, to address prevention and treatment strategies.

Although there is a lack of evidence on the relationship between DF and the quality and duration of sleep, it is known that DFU healing is associated with poor coping and high levels of depression [52], which in turn are associated with poor quality and duration of sleep [36]. To improve the multidisciplinary treatment of patients with DF and psychological disorders or sleep disturbances, research linking these variables should be conducted in the future.

One limitation of this study is that there might be scientific literature published in a different language than those included in the inclusion criteria. In addition, a trend to link sleep disturbances with neuropathic pain has been found in the available literature. However, since neuropathic pain is not exclusive to DF, studies concerning neuropathic pain have not been the topic of this review. The same can be said for PAD, which is multifactorial and not exclusive to DM, and thereforeit has not been considered in the present work either.

## 5. Conclusions

In conclusion, the results suggest that there is a possible association between OSA and the presence or history of DFUs. With respect to sleep quality and duration, no direct associations with DF or DFU variables have been found. It is strongly recommended that future studies, particularly randomized controlled trials, take into account interventions for OSA, sleep quality and sleep duration. These studies should employ highly valid and reliable measurement instruments, which are widely available.

## Figures and Tables

**Table 1:** Structural characteristics of the included studies.

Authors (Year)	Type of Study	Sample Size	Diabetes Type	Gender	Age (Mean ±SD)
Altaf et al. (2016) [24]	Observational CSS	n = 234	2	M= 48 F= 186	Range: 59,6–62,6
Andruskiene et al. (2013) [21]	Observational CSS	n = 1602	-	M= 600 F= 1002	Range: 25–64
Bener et al. (2016) [25]	Observational CSS	n = 459	2	M= 201 F= 258	48,2 ± 9,0 47,1 ± 8,3
Nair (2018) [22]	Observational CSe	n = 100	-	M= 66 F= 34	Range: 18–90
Haveleia and Gayatri (2019) [23]	Observational CSS	n = 97	-	M= 43 F= 54	54,84 ± 1,71
Maltese et al. (2018) [28]	Observational CoS Prospective	n = 94	1 (n = 28) 2 (n= 66)	M= 69% F= 31%	51,5 ± 16,2 62,7 ± 12,0
Puspita et al. (2019) [26]	Observational CSS	n = 152	2	M= 35.5% F= 64.5%	57 ± 8,61
Rutkove et al. (2009) [29]	Observational CoS Prospective	n = 82	1 2	M= - F= -	Range: 18–80
Salomé et al. (2013) [30]	Observational CSS	n = 60	1 (n = 27) 2 (n = 33)	M= 55% F= 45%	Range: 34–71
Sheahan et al. (2017) [27]	Observational C-CS	n = 77	2	M= 50 F= 27	61 ± 11
Subramanian et al. (2019) [20]	Observational C-CS Retrospective	n= 1,656,739	2	M= 902,868 F= 753,871	64.7 ± 13,3
Vas et al. (2016) [19]	Observational CR	Three cases	2	M= 3 F= 0	Range: 57, 61 - 63

Abbreviations: year (Year of publication); SD (Standard Deviation); CoS (Cohort study); C-CS (Case-control study); CSS (Cross-sectional study); CR (Case report); CSe (Case series); M (Male); F (Female).

**Table 2:** Outcome variables related to the sleep and diabetic foot.

Authors (Year)	DF Variables	DF Measurement Tools	SC Variables	SC Measurement Tools
Altaf et al. (2016) [24]	- Diabetic neuropathy - Presence of DFU - Small fiber neuropathy	- Michigan Neuropathy Screening Instrument - PARP activation - Intraepidermalnervefiber density	- Presence of OSA	- Overnight cardio-respiratory device - Apnea/Hypopnea Index
Andruskiene et al. (2013) [21]	- Diabetic foot pain	Unknown	- Problems of falling asleep - Night-time awakenings - Self-rated sleep quality - Sleep latency period - Sleepiness in daytime - Taking naps - Using of sleeping pills	- Basic Nordic Sleep Questionnaire (BNSQ)
Bener et al. (2016) [25]	- Diabetic neuropathy - Presence of DFU	- Observation	- Sleep duration - Sleep loss - Sleep disturbances	- Hours
Nair (2018) [22]	- Inflammatory symptoms - Vasodilation - Gait	- Leg swelling, foot stiffness - Skin discoloration, sensation, leg heaviness,	- Sleep quality	Unknown
Haveleia and Gayatri (2019) [23]	- Presence of DFU - Duration of DFU	Observation	- Sleep quality	- PSQI
Maltese et al. (2018) [28]	- DFU severity - DFU persistence - DFU recurrence	- SINBAD scale - Non-healing in 12-month period - Re-ulceration in a healed site	- Risk of OSA	- STOP-BANG Questionnaire
Puspita et al. (2019) [26]	- Duration of DFU - DFU assessment	- </> 6 months - Wagner scale	- Sleep quality	- PSQI
Rutkove et al. (2009) [29]	- Foot temperature - Nerve conduction studies - Quantitative sensory testing - Diabetic Neuropathy	- iButton - TSA-II NeuroSensory Analyzer - MNSI - UENS	Same as besides, but measurements were done while asleep vs. awake	- iButton - TSA-IINeuroSensory Analyzer - MNSI - UENS
Salomé et al. (2013) [30]	- Presence of DFU	- Observation	- Sleep quality	- PSQI
Sheahan et al. (2017) [27]	- Foot deformity - DFU surface area - DFU infection - DFU depth - Amputation level	- Small muscle wastage, bony prominence, prominent metatarsal heads, hammer/claw toes, limited joint mobility or Charcot deformity - Longest edge and widest edge - University of Texas scale - IWGDF classification	- Daytime sleeping - Lying down duration - Sleep duration	- Epworth Sleepiness Scale - Minutes - Minutes
Subramanian et al. (2019) [20]	- Diabetes-related foot disease	- Signs of amputation, gangrene, presence of DFU, Charcot foot, peripheral vascular disease and peripheral neuropathy	- Presence of OSA	- Previous medical diagnosis
Vas et al. (2016) [19]	- Presence of DFU - Osteomyelitis - DFU healing	- Observation - MRI - Observation	- Presence of OSA	- Previous medical diagnosis

Abbreviations: DF: diabetic foot; SC: sleep; PARP: poly ADP ribosepolymerase; OSA: obstructive sleep apnea; DFU: diabetic foot ulcer; PSQI: Pittsburgh sleep quality index; MNSI: Michigan neuropathy screening index; UENS: Utah early neuropathy scale; MRI: magnetic resonance imaging.

**Table 3:** Findings of selected studies that relate variables of the diabetic foot and variables of the sleep.

Sleep Variable	Diabetic Foot Variable	Findings
OSA	IENFD	- Negative correlation (*p* < 0.001) between IENFD and OSA that implies small fiber neuropathy [24]
	MNSI	- Mild OSA was associated with past history of DFU(*p* = 0.016) [24]
	History of DFU	- Positive correlation with OSA presence (*p* = 0.022) [24]
	PARP	- Positive correlation (*p* = 0.025)between PARP and OSA that involves endothelial dysfunction [24]
	DF presence	- DF was significantly predictive of OSA [20]
	DFU healing	- CPAP therapy for OSA led to DFU healing in patients under treatment [19] - High risk of OSA led to poor DFU healing [28]
Sleep quality	DFU healing	- Microcurrent therapy for DFU led to a significantly better sleep quality [22]
	DFU presence	- Subjective sleep quality showed significant disparity with comprehensive sleep quality (PQSI) [23] - Poor sleep quality was significantly related to pain level (*p* = 0.013) [26] - No significant difference in sleep quality of people with diabetes with and without DFU [26] - Pain (*p*: 0.048) and stress (*p*: 0.001) were significantly related to poor sleep quality [23] - Patients with DFU had poor sleep quality (Salomé et al., 2013)
Sleep duration	Minor amputation presence	- Patients with minor amputation had lower Epworth Sleepiness Scale score (lower score = normal) than those without amputation [27]
	DFU presence	- DFU group showed no differences from DM and/or DN groups in lying down duration and sleep duration [27]

Abbreviations: OSA: obstructive sleep apnea; DFU: diabetic foot ulcer; DF: diabetic foot; CPAP: continuous positive airway therapy; DM: diabetes mellitus; IENFD: intraepidermal nerve fiber density; MNSI: Michigan neuropathy screening instrument;PARP: poly ADP ribose polymerase; PSQI: Pittsburgh sleep quality index.
